# Antibiotic residues in milk and eggs of commercial and local farms at Chittagong, Bangladesh

**DOI:** 10.14202/vetworld.2015.467-471

**Published:** 2015-04-10

**Authors:** Suchayan Chowdhury, Mohammad Mahmudul Hassan, Mahabub Alam, Sarmina Sattar, Md. Saiful Bari, A. K. M. Saifuddin, Md. Ahasanul Hoque

**Affiliations:** 1 Department of Livestock Services, Upazilla Livestock Office, Rangamati Sadar, Rangamati Hill Tracts, Bangladesh; 2 Department of Physiology, Biochemistry and Pharmacology, Faculty of Veterinary Medicine, Chittagong Veterinary and Animal Sciences University, Khulshi, Chittagong-4225, Bangladesh; 3 Department of Animal Science and Nutrition, Faculty of Veterinary Medicine, Chittagong Veterinary and Animal Sciences University, Khulshi, Chittagong-4225, Bangladesh; 4 Department of Dairy and Poultry Science, Faculty of Veterinary Medicine, Chittagong Veterinary and Animal Sciences University, Khulshi, Chittagong-4225, Bangladesh

**Keywords:** antibiotic residues, commercial, eggs, local, milk, thin-layer chromatography, ultra-high-pressure liquid chromatography

## Abstract

**Aim:**

The study was conducted to detection and determination of concentration or level of antibiotic residues in milk and egg of local and commercial farms at Chittagong during December 2011 to June 2012.

**Materials and Methods:**

A total of 400 (200 milk and 200 egg) samples were collected from local and commercial dairy cows and local scavenging and commercial poultry farms, respectively. Microbial inhibition test and thin layer chromatography were used for screening and ultra-high performance liquid chromatography was used to estimate the concentrations of antibiotic residues in samples.

**Results:**

Tetracycline, amoxicillin, and ciprofloxacin residues were significantly (p ≤ 0.05) higher in commercial farms than local. The boiling insignificantly (p>0.05) reduced residues level in milk and egg. The average concentrations of amoxicillin residue in local milk, commercial milk, local egg, and commercial egg were 9.84 µg/ml, 56.16 µg/ml, 10.46 µg/g and 48.82 µg/g, respectively, in raw samples and were reduced to 9.81 µg/ml, 55.54 µg/ml, 10.29 µg/g, and 48.38 µg/g, respectively, after boiling.

**Conclusions:**

Proper maintaining of the withdrawal period and development of active surveillance system are highly recommended for public health security.

## Introduction

The frequent use of antibiotics in clinical practice causes the occurrence of antibiotic residues in various food products of animal origin including milk, egg, and meat. Presence of drugs or antibiotics residues in food above the maximum acceptable level has been recognized worldwide by various public authorities [1]. The antibiotic contamination of milk was reported to be due to intramammary infusions of antibiotics for treating mastitis (92%), injections (6%), and other causes 2% [2]. In case of eggs, the pattern of appearance of drug residues will be influenced by the formation of yolk and white. Presence of antibiotic residues in milk produces difficulties invalidation of certain quality control tests [3]. For human concern, antibiotic residues in food of animal origin produces potential threat to direct toxicity in human (cancers, allergic reactions, etc.), and low levels of antibiotic exposure results in alteration of microflora, and the possible development of resistance [4,5], which cause failure of antibiotic therapy in clinical situations. Protection of public health against possible harmful effect of veterinary drug residues is relatively preoccupation. The initial intention for adequate consumption led the desire to achieve complete elimination of all traces of drug residues in food commodities. Production of safe and quality milk and egg for ensuring better human health is the key aspect of proper public health [3]. To detect antibiotic residues, screening methods and chromatographic techniques were developed. The screening method is generally performed by microbiological, enzymatic, and immunological methods. The earliest screening methods used for detecting antimicrobial residues in foods, including milk were based on the detection of growth inhibition of various bacterial strains evidenced by microbial agar diffusion tests or inhibition of acid production by starter organisms. From the 1950s, assays were developed for the testing of tissues, primarily from the existing milk testing procedures [6]. Although the microbiological assay techniques have been recommended as official and conventional methods because of their simplicity, the bioassay methods lack specificity, and provide only semi-quantitative measurements of residues detected and sometimes produce false positives [7]. Therefore, chromatographic techniques, such as thin layer chromatography (TLC), and high pressure liquid chromatography (HPLC), and capillary electrophoresis (CE), have been developed to replace microbiological assays [8].

From the above facts, it may be mentioned here that due to lack of knowledge and effective dairy and poultry principles in this country, antibiotics are used indiscriminately and withdrawal periods are not being maintained. These antibiotic residues might be a potential hazard for human as well as animal health and a great obstacle to export milk and eggs. In this context, this research work was undertaken to detect and determine concentration or level of antibiotic residues in milk and egg of local and commercial farms in Chittagong district of Bangladesh.

## Materials and Methods

### Ethical approval

The present study was approved by Institutional Animal Ethics Committee.

### Study area and duration

The study was conducted at Raozan Upazila and Chittagong Metropolitan area of Chittagong district during the period of December 2011 to June 2012.

### Milk and egg sample collection

In Raozan Upazila, we selected 10 unions (n = 15) followed by 10 household farms per union and 1 sample per household farm were collected randomly. Accordingly, a total of 100 milk samples were collected from 10 unions. For Chittagong Metropolitan area, we selected 10 commercial farms (n = 150) and 10 milk samples were collected from 10 individual animals of each selected farm randomly. The samples were transferred to the lab through ice box and stored at 4°C until analysis. Same strategy was followed during egg sample collection from scavenging poultry at Raozan Upazila and commercial layer farms in Chittagong Metropolitan area. After homogenization of milk and yolk and albumin of eggs, 5 ml suspension was boiled at 100°C for 20 min.

### Sample evaluation

Both raw and boiled milk and egg samples obtained were primarily evaluated by microbial inhibition test (MIT) to determine the zone of inhibition as described by [9] followed by TLC to detect the presence of residues as described by [10]. Few representative TLC positive samples with the highest grade were analyzed by the ultra HPLC (UHPLC) as described by [3] for determination of concentration or level of antibiotic residues in the samples.

### Statistical analysis

The obtained data were entered in MS Excel-2003 and exported to STATA/IC-11.0 for data analysis. Descriptive analysis was performed for percentages (%) of each variable. Chi-square test and two-sample Wilcoxon rank-sum (Mann–Whitney) test were used to determine the level of significance. The level of significance was set at 0.05.

## Results

### Tetracycline residue

Of 200 milk samples tested by MIT, the residue of tetracycline group was detected in 12% of local cow milk, which was significantly lower than that of commercial cow milk (23%) (p = 0.05). Almost similar results were found in TLC. After boiling the tetracycline residue in local cow milk were 11% (MIT) and 10% (TLC). In commercial dairy milk, the presence of tetracycline residue was 23% (MIT) and 21% (TLC) after boiling.

Boiling the samples had not changed the earlier result for antibiotic residues obtained by MIT (p = 0.82) and TLC (p = 1.00). On TLC testing, a little variation was observed on MIT testing result regardless of un-boiled and boiled samples (Table-1).

**Table-1:** Comparison of tetracycline residues in milk samples between local and commercial cows.

Sample (Milk)	MIT (%)			TLC (%)		
	
	Before boil	After boil	p-value	Before boil	After boil	p-value
Local cows	12 (12)	11 (11)	0.82	10 (10)	10 (10)	1.00
Commercial dairy cow	23 (23)	23 (23)	1.00	23 (23)	21 (21)	0.86
p-value	0.05	0.03		0.02	0.05	

n=100, MIT=Microbial inhibition test, TLC=Thin layer chromatography

Of 200 egg samples tested by MIT, the residue of antibiotic of tetracycline group were detected in 7% local egg, 25% in commercial egg, 5% in boiled local egg, and 24% in boiled commercial egg samples (Table-1). On the other hand, samples evaluated in TLC testing tetracycline positive sample of local egg, commercial egg, boiled local egg, and boiled commercial egg were 7%, 25%, 4%, and 24%, respectively (Table-2).

**Table-2:** Comparison of tetracycline residues in egg samples between local and commercial hens.

Sample (Egg)	MIT (%)			TLC (%)		
	
	Before boil	After boil	p-value	Before boil	After boil	P value
Local scavenging hens	7 (7)	5 (5)	0.76	7 (7)	4 (4)	0.81
Commercial layers	25 (25)	24 (24)	0.86	25 (25)	24 (24)	0.86
p-value	0.001	0.001		0.001	0.001	

n=100, MIT=Microbial inhibition test, TLC=Thin layer chromatography

### Amoxicillin residue

Among 200 milk samples analyzed, Amoxicillin residue was detected in raw local milk (14%), raw commercial milk (38%), boiled local milk (12%), boiled commercial milk (37%). In TLC testing, Amoxicillin was also detected in raw local milk (13%), in raw commercial milk (35%), in boiled local milk (12%), and in boiled commercial milk (35%) as shown in Table-3. The frequency of amoxicillin residue was significantly greater in the sample of commercial cow’s than the samples of local cow’s milk regardless of diagnostic tests, boiled (p = 0.0002), and un-boiled (p = 0.0002) samples.

**Table-3:** Comparison of Amoxicillin residues in milk samples between local and commercial cows.

Sample (Milk)	MIT (%)			TLC (%)		
	
	Before boil	After boil	p-value	Before boil	After boil	p-value
Local cows	14 (14)	12 (12)	0.82	13 (13	12 (12)	0.81
Commercial dairy cow	38 (38)	37 (37)	0.87	35 (35)	35 (35)	1.00
p-value	0.0002	0.0002		0.0005	0.0002	

n=100, MIT=Microbial inhibition test, TLC=Thin layer chromatography

Of 200 egg samples tested using MIT the residue of Amoxicillin were more frequent in commercial eggs (17%, both for raw and boiled sample) than others. Almost identical results were recorded in these samples tested by TLC (Table-4) regardless of diagnostic types and raw and boiled samples. The residue detection was more common in commercial egg samples than in local egg samples (p = 0.01 and p = 0.002).

**Table-4:** Comparison of residue of Amoxicillin in egg samples between local and commercial hens

Sample (Eggs)	MIT (%)			TLC (%)		
	
	Before boil	After boil	p-value	Before boil	After boil	p-value
Local scavenging hens	5 (5)	3 (3)	0.71	5 (5)	4 (4)	0.73
Commercial layers	17 (17)	17 (17)	1.00	17 (17)	15 (15)	0.79
p-value	0.01	0.002		0.01	0.01	

n=100, MIT=Microbial inhibition test, TLC=Thin layer chromatography

### Ciprofloxacin residue

Of 200 milk samples tested by MIT the residues of Ciprofloxacin were dominant in commercial milk sample (17% raw and 16% boiled) (p = 0.84) than local cow milk (8% raw and 5% boiled) (p = 0.56). The results of both the MIT and TLC were almost the same (Table-5).

**Table-5:** Comparison of ciprofloxacin residues in milk samples between local and commercial cows.

Sample (Milk)	MIT (%)			TLC (%)		
	
	Before boil	After boil	p-value	Before boil	After boil	p-value
Local cows	8 (8)	5 (5)	0.56	7 (7)	5 (5)	0.76
Commercial dairy cow	17 (17)	16 (16)	0.84	15 (15)	14 (14)	0.83
p-value	0.08	0.02		0.11	0.05	

n=100, MIT=Microbial inhibition test, TLC=Thin layer chromatography

A total of 200 egg samples tested in MIT the residue of Ciprofloxacin were detected in 9% of local egg, 45% in commercial egg, 6% in boiled local egg, and 43% in boiled commercial egg. On the other hand, in TLC, the residue of tetracycline positive sample of local egg, commercial egg, boiled local egg, and boiled commercial egg were 9%, 45%, 7%, and 44%, respectively (Table-6). The level of ciprofloxacin residue was significantly higher in commercial eggs than in local eggs regardless of diagnostic test and types of treatment (p = 0.0001).

**Table-6:** Comparison of ciprofloxacin residues percentages in egg samples before and after boiling.

Sample (Egg)	MIT (%)			TLC (%)		
	
	Before boil	After boil	p-value	Before boil	After boil	p-value
Local scavenging hens	9 (9)	6 (6)	0.59	9 (9)	7 (7)	0.79
Commercial layers	45 (45)	43 (43)	0.88	45 (45)	44 (44)	0.88
p-value	0.0001	0.0001		0.0001	0.0001	

n=100, MIT=Microbial inhibition test, TLC=Thin layer chromatography

### Comparison of amoxicillin residue in milk by UHPLC

The average concentration of amoxicillin residue was significantly higher in commercial raw milk (56.16 mg/ml) than local raw milk (9.84 mg/ml) (p = 0.01). After boiling, the average concentration of amoxicillin was reduced a very little both in commercial milk (55.54 mg/ml) and local milk (9.8 mg/ml) as illustrated in Table-7.

**Table-7:** Comparison of Amoxicillin residues in milk samples between local and commercial cows.

Sample (Milk)	Average concentration (µg/ml)		

	Before boil	After boil	p-value
Local cows	9.84	9.81	0.20
Commercial dairy cow	56.16	55.54	0.20
p-value	0.05	0.05	

n=5

### Comparison of Amoxicillin residue in egg by UHPLC

The average concentration of amoxicillin residue in commercial layer (48.82 mg/g) was significantly higher than local scavenging hens (10.46 mg/g) (p = 0.03). After boiling the samples, the same results retained regardless of local and commercial eggs (Table-8).

**Table-8:** Comparison of conc. of Amoxicillin residues in egg samples between local and commercial hens.

Sample (Egg)	Average concentration (µg/ml)		

	Before boil	After boil	p-value
Local scavenging hens (n=5)	10.46	10.29	0.37
Commercial layers (n=5)	48.82	48.38	0.21
p-value	0.04	0.05	

## Discussion

The antibiotic residues were more frequently found in commercial dairy and layer farms. The antibiotic residues did not reduce markedly by boiling. The concentration of antibiotic residues determined by UHPLC was higher than minimum residue level. It is alarming for public health safety.

The ciprofloxacin residues detected in commercial layer eggs was more frequent than other antibiotic under study. It was due to the random use of ciprofloxacin in the treatment of diseases of commercial layers. Boo *et al.*, [11] also found the similar results.

Amoxicillin residues were found more frequent in the milk of commercial dairy cows. The present result is supported by [12,13]. Amoxicillin is commonly used for treating mastitis, but most of the time withdrawal period is not maintained. It may cause the presence of amoxicillin residues in milk. Luboslava *et al.*, [14] identified amoxicillin residues in table eggs and poultry meat [15].

Tetracycline residues were found more in commercial layers. The present finding is agreed by [16]. It may be due to the use of tetracycline in feed as the growth promoter and treating of layer. Tetracycline residues were also high in commercial milk. Mehran *et al.*, [17] also found a similar result. It may be due to use of tetracycline in the treatment of various bacterial diseases in the dairy farms. The effect of boiling on antibiotic residue was not significant. However, it reduced lower amount of tetracycline residues in raw milk samples. It might be due to heat processing effect on drug residues. Further processing of milk can bring on the lowering on the concentrations of tetracycline and other antibiotics.

In the present study, it has been found that milk collected from commercial dairy farm and eggs from commercial layer farms has higher concentration of residues of tetracyclines and ciprofloxacin, respectively. This is might be due to indiscriminate use and improper monitoring system for antibiotic withdrawal period of specific antibiotics in the commercial dairy and layer farms. Hence, the percentage of residue of antibiotics is high in commercial dairy and layer farms in comparison of local farms.

It is known that drugs residue on animal products especially milk and egg is an important problem in most countries. Analytical thin layer chromatography which employed in our study was not tasked for measuring the concentration of antibiotic residues, but for detection their presence in dairy milk and chicken eggs. This technique is simple, exact, and non-expensive, which can be executed easily in most laboratories, but with less accuracy than HPLC.

Many countries of the world regulate drugs use in food producing animals and set up limits. Tolerance level of amoxicillin residues according to U.S. official is 0.01 ppm (10 ppb or 10 mg/kg) in milk and uncooked edible tissues of cattle [18]. Presence of antibiotic residues above tolerance level induced allergic reaction which is a potential risk for public health safety. Indiscriminate and irrational use of antibiotics in livestock without following withdrawal period may result in unexpected residues in food supplies and could cause serious health hazards to consumers.

It was observed that the concentration of amoxicillin residue in milk of local cow ranges from 4.87 to 12.60 mg/ml milk. Where commercial dairy farms milk ranging 8.49-180.12 mb/ml, 7.3-15.87 mg/g for local eggs, 14.76-168 mg/g for commercial eggs. After boiling the residue concentrations decrease average 0.03 mg/ml for local milk, 0.62 mg/ml for commercial milk, 0.17 mg/g for local hen’s eggs, 0.44 mg/g for commercial eggs. There were no previous data available of concentration or level of antimicrobial residues in our country on milk and eggs by UHPLC but has some observation on meat [15]. It is clear from the result is that antibiotics already exist in our food chain, especially in milk and eggs. The awareness regarding to residue testing should be increased to determine the exact level of antibiotic residue in food for ensuring public health safety. It is therefore important that their use of food animals be done with utmost care; antibiotics should be given at recommended doses and with appropriate supervision. Adequate withdrawal period should be observed in all milking cows and laying hens following therapeutic use of antibiotics.

## Conclusion

The presence antibiotic residue percentages in milk and eggs were higher in commercial dairy and layer farms in comparison of local farms. Boiling slightly reduce the antibiotic residues in both raw and boil milk and eggs. Indiscriminate and irrational use of antibiotics in livestock without following withdrawal period may result in unexpected residues in food supplies and could cause serious health hazards to consumers. Adequate withdrawal period should be observed in all milking cows and laying hens following therapeutic use of antibiotics. Actually, the use of antibiotics in food animals by Non-Veterinarian should be discouraged. Both veterinarians and food producers should become extremely conscious of the need for high public confidence in the products they produce. This necessitates that all efforts including awareness creation, observance of the withdrawal period, effective surveillance, monitoring, and control on the use of veterinary drugs to prevent drug residues in animal derived products be employed.

## Authors’ Contributions

SC, MMH, and MAH planned and implemented the study design and carried out the laboratory experimentation. MMH, MA, AKMS, SS, MSB and MAH drafted and revised the manuscript. All authors read and approved the final version of the manuscript.
